# The relationship between moral distress, ethical climate, and attitudes towards care of a dying neonate among NICU nurses

**DOI:** 10.1186/s12912-023-01459-7

**Published:** 2023-09-05

**Authors:** Zeinab Rezaei, Monirsadat Nematollahi, Neda Asadi

**Affiliations:** 1 Department of Pediatric and Neonatal Intensive Care Nursing, Kerman University of Medical Sciences, Kerman, Iran; 2 Nursing Research Center, Kerman University of Medical Sciences, Kerman, Iran

**Keywords:** Moral distress, Ethical climate, Attitude, Dying, NICU

## Abstract

**Background:**

Nurses working in neonatal intensive care units play a crucial role in providing care to critically ill or premature neonates. However, is not without its challenges, particularly when it comes to making difficult ethical decisions about end-of-life care. In some cases, neonates do not survive despite the best efforts of medical professionals. The present study aimed to investigate the relationship between moral distress, ethical climate, and attitudes towards end-of-life care among nurses working in neonatal intensive care units.

**Methods:**

This is a descriptive-analytical cross-sectional study (May 21, 2021).The research population included 126 nurses working in neonatal intensive care units in Kerman province (Kerman, Jiroft, Bam, and Rafsanjan). Data collection tools included four questionnaires: demographic information, the Frommelt Attitudes towards Care of the Dying (FATCOD), the Hospital Ethical Climate Survey, and the Moral Distress Scale. SPSS22 was used to analyze the data.

**Results:**

The results revealed that the mean frequency and intensity of moral distress were 44.42 ± 17.67 and 49.45 ± 17.11, respectively. The mean ethical climate was 92.21 ± 17.52 and the FATCOD was 89.75 ± 9.08, indicating NICU nurses’ positive perceptions of ethical climate and their favorable attitudes towards EOL care, respectively. The results showed a direct and significant relationship between ethical climate and the FATCOD (P = 0.003, r = 0.26).

**Discussion:**

We suggest policymakers and managers design strategies for better ethical climate in hospitals and reduction of moral distress among nurses.

## Introduction

Many neonates need admissions to neonatal intensive care units (NICU) for various reasons [1], but not all of them survive, and some require end-of-life)EOL) care [2]. According to UNICEF, 2.4 million neonates died in 2019 (6,700 neonatal deaths every day). Shockingly, one-third of these deaths occurred on the day after birth, and three-quarters occurred within the first week after birth [3]. Therefore, EOL care is an integral part of nursing care in NICUs that provides comfort, dignity, and necessary support for neonates and their families [4].

EOL care is a crucial aspect of palliative care that provides comfort, dignity, and support for both neonates and their families [5]. Despite the fact that about two-thirds of neonatal deaths occur in the NICU [6], infants receive less EOL care than adults [7]. This is partly due to the fragmented and inconsistent use of palliative care in clinical settings, where protocols and therapeutic measures are often prioritized over compassionate EOL care [8].

One of the most essential components of EOL care for neonates is assessing and addressing distressing symptoms. There is limited evidence to guide neonatal EOL symptom management and therefore significant variety in treatment. EOL neonatal palliative care should include identifying and relieving distressing symptoms. Symptoms to manage at neonatal EOL may include pain using both non-pharmacologic and pharmacologic comfort measures, respiratory distress, secretions, agitation and neurologic symptoms, nutrition and gastrointestinal distress, and skin care. Also of equal importance is communication surrounding familial existential distress and psychosocial care. Institutions should implement a guideline for neonatal EOL care as guidelines have been shown to decrease variability of interventions and increase use of pharmacologic symptom management [9].

As the main members of the healthcare team, nurses play a crucial role in providing EOL care to neonates in NICUs. They rely on a combination of knowledge, attitudes, beliefs, and experiences to provide compassionate and ethical care to their patients [10, 11]. Some NICU nurses believe that prolonged EOL care may actually increase neonatal suffering, and they may view unnecessary painful procedures and laboratory tests as tortures [7]. These beliefs can cause significant moral distress for nurses, who may feel that they are unable to act in accordance with their ethical values.

EOL care for dying neonates can affect moral distress of the nurses [8]. Moral distress is not the same as discomfort with a medical procedure to which a healthcare professional may be morally opposed such as sex reassignment surgery or abortion. Rather, moral distress occurs when professionals feel that they are unable to act in accordance with their ethical values (e.g. Avoiding inappropriate treatment, minimizing unnecessary suffering, telling the truth) [12]. Studies have shown that NICU nurses experience more moral distress during EOL care than nurses in other wards [10].

Some studies indicated an inverse relationship between moral distress and ethical climate. Ethical climate refers to the perceptions of the work environment related to the management of moral issues and communication between healthcare professionals [13, 14]. Nurses’ perception of ethical climate is an important factor in determining moral distress [15]. Given the complex relationship between ethical issues and EOL care, it is essential to explore the relationship between moral distress, ethical climate, and attitudes towards EOL care among NICU nurses.

## Methods

### Study design and setting

This is a descriptive-analytical cross-sectional study (May-July 2021). The research population consisted of 130 nurses working in the NICUs of Kerman province, indicating the cities of Kerman, Jiroft, Bam, and Rafsanjan. Inclusion criterion was participants’ bachelor’s degree in nursing. The exclusion criterion was failure to respond to more than 10% of the questions.

### Sample size and sampling

Due to the limitation of the statistical population, the research sample was equivalent to the research population. The sample size of the study was based on the census of nurses working in the NICUs of hospitals affiliated to Kerman University of Medical Sciences. A total of 130 nurses working in these departments were willing to participate in the study, and the questionnaires were distributed among them. Out of this number, four questionnaires were removed because more than 10% of the questions were unanswered, leaving 126 questionnaires for analysis.

### Instrument

Data were collected using four questionnaires, including socio-demographic form, the Moral Distress Scale, the Hospital Ethical Climate and Frommelt Attitudes towards Care of the Dying (FATCOD).

#### Socio-demographic form

this form includes gender, age, marital status, educational level, type of employment, work experience, NICU working experience, monthly income, receiving education about EOL care and history of caring for a dying infant.

#### Moral Distress Scale (MDS)

This questionnaire was originally developed by Corley et al. [16] and used in many domestic and foreign studies. In Iran, Joolaee et al. made changes to the questionnaire in 2013 to reflect the conditions of providing services in Iran and to address the existing problems in Iranian hospitals. The adapted version was validated for content and reliability, with a Cronbach’s alpha of 0.86 [16]. The questionnaires were completed by 15 participants twice with a 2-week interval. To determine internal consistency, Cronbach’s alpha coefficients were found to be 0.87. MDS contains 24 items, each relating to a particular situation in hospital care. This scale examines nurses’s understanding of two aspects: “severity of moral distress” and “frequency of stressful situations faced by the individual.” In this scale, the nurses specify how frequently they face stressful situations that may cause moral distress in them. It also assesses how intense each of these stressful situations is. This tool has two 5-point Likert scales: one for frequency of moral distress (from never = 0 to very much = 4) and the other for severity of moral distress (from causing no distress = 0 to causing severe distress = 4), with score range from 0 to 96. Higher scores indicate higher intensity and frequency of moral distress.The division of intensity and frequency of moral tension in this questionnaire is as follows: 0–31 indicates low intensity or frequency of moral tension, 32–64 indicates moderate intensity or frequency of moral tension, and 65–96 means high intensity or frequency of tension [17].

#### Hospital ethical climate survey

The second part of the study used Olson’s Ethical Climate Scale, which was originally designed by Olson in 1995 to measure hospital ethical climate. Olson determined the validity of the questionnaire using content validity index (CVI) at 87% and its reliability at 91% using Cronbach’s alpha [18]. This scale was later translated and used in a study by Hariri et al. in 2011 [19], where its content validity index and reliability were measured using internal consistency and test-retest methods (content validity was 0.89). In the present study, the translated tool from Hariri’s study was used. This scale consists of 26 items scored on a five-point scale, ranging from almost never [1] to almost always [5]. The score of each questionnaire ranges between 26 and 130, with higher scores indicating more positive ethical climates. This questionnaire contains five factors that evaluate nurses’ perceptions of their colleagues (questions 1, 10, 18and 23), patients (questions 2, 6, 11 and 19), managers (questions 3, 7, 12, 15, 20 and 24), hospital (questions 4, 8, 13, 16, 21 and 25), and physicians (questions 5, 9, 14, 17, 22 and 26).

#### Frommelt Attitudes towards Care of the dying (FATCOD)

The questionnaire was designed by Frommelt et al. in 1991 [20] and can be used for patients in the final stages of any age and consists of 30 items. Fifteen of these items are expressed positively [1–2–4–10–12–16–18–19–20–21–22–23–24–25–29] and relate to nurses’ attitudes towards the role of the family in the care of dying patients. The other 15 items are expressed negatively [3, 5, 6, 7, 8, 9, 11, 13, 14, 15, 17, 26, 27, 28 and 30] and relate to the fears and stresses of nurses in caring for dying patients.

To organize the scores of the questionnaire, a Likert scale is used, with responses ranging from I strongly disagree to I strongly agree. For positive questions, a score of five is assigned to I strongly disagree, while for negative questions, scoring is done in reverse. Therefore, the range of scores is between 30 and 150 [21]. The validity and reliability of this questionnaire have been investigated and confirmed in previous studies [20]. Frommelt et al. reported a Cronbach’s alpha of 0.76, which indicated an acceptable level of reliability [22].

### Data collection and ethical consideration

In the present study, the target population consisted of NICU nurses in educational hospitals of Kerman province. After obtaining the IRB approval from the ethics committee of Kerman University of Medical Sciences (IR.KMU.REC.1400.349), the researcher referred to the educational hospitals, and explained participants the study purpose and method. The researchers visited the relevant centers to collect data during all three shifts of morning, evening, and night. The questionnaires were provided to the nurses when the workload of the ward was less and they were asked to complete them carefully. The researcher went to the hospital the next day to collect the completed questionnaires. Samples were assured that their responses would remain confidential.

### Data analysis

SPSS 22 was used to analyze the data. The data were described using descriptive statistics (frequency, percentage, mean, and standard deviation). As the main variables of the study had normal distribution, the Pearson Correlation Coefficient was used to assess the correlation between moral distress, ethical climate, and the FATCOD. The demographic information of the research samples was analyzed using independent t-test and ANOVA. The significance level of 0.05 was used.

## Results

The study analyzed 126 questionnaires and showed that the mean age of the nurses was 32.21 ± 6.89, and their work experience was 7.57 ± 5.57 years. Most of the nurses were married (78.6%), had a bachelor’s degree (90.5%), were hired nurses or contract recruiters, had unfixed shifts (95.2%), and had income above 5 million Tomans (89.7%). The majority of the nurses did not participate in EOL care courses (65.1%), and experienced caring for a dying neonate (91.3%).

The mean frequency and intensity of moral distress were 44.42 ± 17.67 and 49.45 ± 17.11, respectively, indicating moderate frequency and intensity of moral distress among the NICU nurses. The mean ethical climate score was 92.21 ± 17.52, indicating the positive ethical climate prevailing in the NICUs and the mean FATCOD score was 89.75 ± 9.08, indicating favorable attitude towards EOL care (Table 1).

**Table 1:** Mean and standard deviation of moral distress, ethical climate, and FATCOD in the NICU nurses

Variable	Mean ± SD
Frequency of moral distress	44.42 ± 17.67
Intensity of moral distress	49.45 ± 17.11
Ethical climate	92.21 ± 17.52
FATCOD	98.75 ± 9.08

The results showed a direct and significant relationship between ethical climate and the FATCOD, meaning that the more positive the ethical climate, the more positive the nurses’ attitudes towards neonatal EOL care (P = 0.003, r = 0.26) (Table 2).

**Table 2:** The relationship between moral distress and FATCOD in the NICU nurses

FATCOD	Correlation coefficient	P-value*
Frequency of moral distress	0.12	0.190
Intensity of moral distress	0.16	0.079
Ethical climate	0.26	0.003

Table 3 presents a significant and inverse relationship between the intensity of moral distress and the ethical climate. This suggests that as the intensity of moral distress increases, the ethical climate becomes less favorable (r=-0.19), (p = 0.03). In addition, the study found a significant and inverse relationship between moral distress and ethical climate in colleagues (p = 0.02), patients (p = 0.01) and nurse managers (p = 0.01). The intensity of moral distress in colleagues (r=-0.20), patients (r=-0.22), and nurse managers (r=-0.21) decreased.

**Table 3:** The relationship between moral distress and ethical climate in the NICU nurses

Variable		Correlation coefficient	P-value*
The number of times faced with moral tension	Ethical climate of colleagues	-0.17	0.06
The intensity of moral distress		-0.20	0.02
The number of times faced with moral tension	Ethical climate doctors	-0.07	0.46
The intensity of moral distress		-0.07	0.43
The number of times faced with moral tension	Ethical climate doctors	-0.13	0.18
The intensity of moral distress		-0.13	0.14
The number of times faced with moral tension	Ethical climate patients	-0.04	0.68
The intensity of moral distress		-0.22	0.01
The number of times faced with moral tension	Ethical climate nurse managers	-0.05	0.56
The intensity of moral distress		-0.21	0.01
The number of times faced with moral tension	Total ethical climate	-0.10	0.24
The intensity of moral distress		-0.19	0.03

Table 4 indicated a significant difference in nurses’ moral distress based on their education level, so nurses with master’s degree experienced more moral distress than nurses with bachelor’s degree (P = 0.005). We found a significant difference in the mean ethical climate based on employment status, monthly income, and participation in EOL care courses (P > 0.05), so hired nurses reported a more favorable ethical climate. Nurses whose incomes were more than 5 million Tomans per month, as well as those who participated in EOL care courses reported a more positive ethical climate.

**Table 4:** The relationship between the demographic variables, moral distress, ethical climate, and FATCOD among the NICU nurses

Variable	Group	Mean ± SD of moral distress	Statistic test, P- value	Mean ± SD of ethical climate	Statistic test, P value	Mean ± SD of FATCOD	Statistic test, p value
Marital status	Single	46.48 ± 17.64	T = − 1.01 P = 0.311	97.07 ± 16.98	T = 1.64 P = 0.104	90.41 ± 8.66	T = 0.42 P = 0.675
	Married	50.26 ± 16.97		90.88 ± 17.51		89.58 ± 9.23	
Education level	Bachelor’s degree	48.07 ± 16.34	T=- 2.86 P = 0.005	91.92 ± 18.05	T = − 0.56 P = 0.575	89.57 ± 9.30	T = − 0.66 P = 0.507
	Master’s degree	62.50 ± 19.48		92.92 ± 11.45		91.41 ± 6.71	
Employment	Hired	50.85 ± 17.32	T = 1.40 P = 0.245	104.43 ± 15.48	F = 6.44 P < 0.001	88.46 ± 9.58	F = 1.27 P = 0.286
	Contract recruiter*	53.95 ± 18.14		87.77 ± 14.40		89.77 ± 7.02	
Position	Nurse	49.04 ± 17.31	T = − 0.70 P = 0.487	92.37 ± 17.63	T = 0.67 P = 0.494	89.76 ± 9.18	T = 0.08 P = 0.936
	Head nurse	53.95 ± 18.14		85.33 ± 12.34		89.33 ± 3.79	
Type of shift	Unfixed	49.04 ± 17.14	T = − 1.21 P = 0.230	92.47 ± 17.39	T = 0.74 P = 0.458	89.86 ± 9.23	T = 0.62 P = 0.536
	Fixed	57.67 ± 15.64		87.00 ± 21.09		87.50 ± 5.31	
Income	< 5 million Tomans	42.61 ± 12.12	T = − 1.53 P = 0.129	105.92 ± 18.93	T = 3.08 P = 0.003	88.77 ± 8.80	T = − 0.41 P = 0.682
	> 5 million Tomans	50.23 ± 17.47		90.63 ± 13.73		89.87 ± 9.15	
Participation in EOLcare courses	Yes	51.84 ± 18.66	T = 1.15 P = 0.253	96.57 ± 18.54	T = 2.07 P = 0.040	90.81 ± 6.18	T = 0.96 P = 0.338
	No	48.17 ± 16.19		89.87 ± 16.59		89.18 ± 10.30	
Experience of caring for a dying neonate	Yes	49.63 ± 17.29	T = 0.39 P = 0.701	91.74 ± 17.78	T = 1.74 P = 0.335	89.84 ± 17.29	T = 0.36 P = 0.722
	No	47.54 ± 15.78		97.09 ± 14.29		88.81 ± 15.78	

* A contract RN is someone who completes all the duties and responsibilities of a nurse but is contracted for a temporary amount of time

## Discussion

The present study aimed to investigate the relationship between moral distress, ethical climate and attitudes towards care of a dying among NICU nurses. The study results showed that the intensity and frequency of moral distress among the NICU nurses were moderate.

These results are consistent with previous studies conducted in Iran by Bayat et al. (2019), which also reported moderate levels of moral distress among nurses in selected hospitals in Isfahan and Tehran [23]. However, the results of the present study differ from the findings of other studies, such as those conducted by Habibzadeh et al. (2020) and Wenwen et al. (2018), which reported low levels of moral distress among nurses in intensive care units [24, 25]. This difference in results may be due to differences in research populations and the tools used.

Moral distress is a complex phenomenon that can result from various factors in healthcare settings. In intensive care settings, factors such as understaffing, the need for timeliness and efficiency [29], and situations involving perceived futile care or differing perspectives on EOL decisions can increase the risk of moral distress among healthcare providers [26]. Structural aspects of the NICU, including the lack of a continuous care team, poor communication, and staff shortages can also contribute to moral distress among nurses. These factors can lead to the perception of substandard care being provided [27]. Additionally, diversity in the views and values of providers within an organization can lead to reports of greater ethical climate [28]. When providers on a care team have different values and moral compasses, it is more likely that some team members will feel moral constraint and subsequently moral distress based on the care provided [29].

The present study found a positive ethical climate and a favorable attitude towards EOL care. Cerit and Özveren (2019) reported a moderate level of moral distress and attitude towards ethical climate in Turkish nurses, so a suitable ethical climate significantly reduced moral distress of the nurses [30]. However, the findings of the present study differ from the results of Constantina et al.‘s (2019) study, which showed that nurses had a poor understanding of ethical climate [34]. This difference in results may be due to several factors, including differences in the geographical and working conditions of the nurses in the two studies. A poor ethical climate can exacerbate issues such as moral distress, inadequate or futile care, and failed or inadequate support from others, and may create false hope for patients and their families [23].

Khanjari (2018) supported our results and found a positive attitude towards EOL care among nurses. Abate et al. (2018) and Lancester et al. (2017) also indicated the positive attitude of nurses towards EOL care [31, 32]. Shi et al. (2019) showed that community health care providers had a positive attitude towards EOL care, but lacked professional and systematic knowledge and skills in caring for EOL patients [33]. In addition, previous research has shown that patients receiving primary palliative care have longer survival and a better prognosis compared to patients receiving standard care [34–36]. These findings emphasize the importance of EOL care and the need to strengthen the knowledge and skills of healthcare providers in this area.

The results showed a direct and significant relationship between ethical climate and the FATCOD, indicating that a more positive ethical climate was associated with a more positive attitude towards neonatal EOL care among NICU nurses. These findings are consistent with previous research in this area. Chessa and Moreno (2019) indicated that ethical climate had a significant impact on the quality of EOL care [37] another study found a significant relationship between ethical climate and EOL palliative care [38]. Which is in line with the results of the present study, However, the findings of the present study differ from the results of Lokker et al.‘s (2018) study, which indicated that nurses experienced an unfavorable ethical climate when providing palliative care and reported a significant negative correlation between ethical climate and EOL care of the dying neonates [39]. Researchers concluded that poor EOL care could increase adverse ethical climate [40]. These contradictory results highlight the complexity of ethical issues in EOL care and the effect of several factors on them; Kadivar et al. (2014) considered the ethical issues related to EOL care as one of the most important challenges in NICUs [41].

The study results showed a significant and inverse relationship between the intensity of moral distress and ethical climate. The findings of various studies on nurses working in different clinical environments showed that a favorable ethical climate reduced moral distress [42]. For example, Yujeong et al. (2019) showed that ethical climate had a negative correlation with moral distress in clinical nurses working in the neonatal intensive care unit [43], while Fazl Jo et al. (2017) showed a direct negative relationship between the severity of perceived moral distress and the ethical climate in nurses [44]. Several studies have found that a positive ethical climate and cooperative work environments can reduce moral distress [45, 46]. However, a study by Asgari et al. (2019) contradicts the results of the present study, showing no relationship between ethical climate and the frequency and severity of moral distress among operating room staff [47]. One possible reason for this difference in the results is that special care departments are more sensitive than other departments due to the hospitalization of patients with acute and special conditions. Nurses working in these departments may experience higher levels of moral stress and may require a work environment that is more closely aligned with ethical behavior.

We also investigated the relationship between ethical climate, moral distress, attitude towards EOL care, and demographic variables among NICU nurses and found that nurses with master’s degrees experienced more moral distress. Another research supported our results and indicated that there was no high quality training to prevent moral distress in nurses [48]. Atashzadeh-Shoorideh et al. (2021) found that people’s technical knowledge was insufficient to prevent moral distress and that ethics education was necessary for curricular development [49]. Hough (2008) pointed out that insufficient ethics training left nurses confused and unable to make useful decisions [50]. Mendel (2014) studied strategies to reduce the moral distress of NICU nurses in American hospitals and concluded that improving palliative services could reduce the moral distress of nurses when dealing with neonatal EOL care. In Iran, the undergraduate nursing curriculum only includes 2–4 h of theory about death and its moral issues. Therefore, the educational program in academic centers should be improved to increase nurses’ attitudes towards EOL care.

Hired nurses in this study reported a more favorable ethical climate compared to contract recruiters. Zhang et al. (2019) similarly found a significant relationship between ethical climate and employment status, with contract recruiters reporting a less favorable ethical climate than hired nurses. It is noteworthy that hired nurses receive the same salary and benefits as contract recruiters, but they have higher job security, which may contribute to their better perception of the ethical climate [51].

Monthly income and participation in EOL care courses were among the factors that had an impact on nurses’ positive perception of the ethical climate. To explain the relationship between the ethical climate and income, we must consider various factors affecting the ethical climate. Joolaee et al. (2011) showed that job satisfaction could positively improve the ethical climate and that the income level was one of the factors affecting job satisfaction [16]. Therefore, we found a direct relationship between nurses’ income level and their perception of the ethical climate. Hasani et al. (2017) indicated a direct and significant relationship between ethical climate and job satisfaction among nurses at Imam Khomeini hospital in Urmia [52]. Shafiei et al. (2018) also confirmed these results. In general, the studies indicated that the best predictor of people’s job satisfaction depended on their perceptions of the paid benefits and compensations relative to their individual merit (53).

According to the study results, participation in EOL care courses could affect nurses’ positive perception of the ethical climate. Zhang et al. (2019) supported this finding and indicated that nurses who participated in EOL care courses had a better perception of the ethical climate [51]. These results can be due to professional views individuals acquire through specialized training, which can change their expectations. Researchers revealed that perception of the values and ethics governing the work environment had a significant relationship with training [52].

### Limitation

This study had limitations. Among other things, some research units showed insufficient cooperation or non-cooperation in answering questionnaire questions due to boredom or busy work. Another limitation was the impossibility of face-to-face sampling in the cities, which made the sampling process difficult. In addition, the limited research community is another limitation of the present study. Therefore, there is a need to conduct more and deeper studies such as qualitative methods with a larger sample size on other nurses working in intensive care units.

## Conclusion

This study highlights the close relationship between moral distress, ethical climate, and attitudes towards EOL care among NICU nurses. It is clear that developing strategies to reduce the intensity and frequency of moral distress is one of the important issues in this department. The results of the present study showed the importance of creating a positive moral atmosphere to reduce moral distress in nurses and thus provide better EOL care for infants. We suggest that policymakers and managers develop strategies to improve the ethical climate in hospitals and reduce moral distress among nurses, so that they can establish a compassionate communication with families, reduce their moral distress, and provide quality EOL care for neonates.

## Data Availability

The datasets generated and/or analysed during the current study are not publicly available, but are available from the corresponding author on reasonable request.

## References

[1] Keir A, McPhee A, Wilkinson D (2014). Beyond the borderline: outcomes for inborn infants born at ≤ 500 grams. J Paediatr Child Health.

[2] Kilcullen M, Ireland S (2017). Palliative care in the neonatal unit: neonatal nursing staff perceptions of facilitators and barriers in a regional tertiary nursery. BMC Palliat care.

[3] Namnabati M, Farzi S, Ajoodaniyan N (2016). Care challenges of the neonatal intensive care unit. Iran J Nurs Res.

[4] Boan Pion A, Baenziger J, Fauchère J-C, Gubler D, Hendriks MJ. National Divergences in Perinatal Palliative Care Guidelines and Training in Tertiary NICUs. Front Pead. 2021:678. 10.3389/fped.2021.673545 PMC8316587 34336737

[5] Catlin A, Brandon D, Wool C, Mendes J (2015). Palliative and end-of-life care for newborns and infants: from the National Association of neonatal nurses. Adv Neonatal Care.

[6] Khazani S (2013). Nurses’ perception of actual and ideal organizational ethical climate in hospitals of Ahwaz Jondishapour University of Medical Sciences in 1390-91. Iran J Med Ethics History Med.

[7] kulmala J. Ethical Decision Making and Moral Distress In Nursing Practice A Literature Review. Bachelor’s thesis. Degree Programme. 2016.

[8] Mendel TR (2014). The use of neonatal palliative care: reducing moral distress in NICU nurses. J Neonatal Nurs.

[9] Lewis SL (2019). Emotional intelligence in neonatal intensive care unit nurses: decreasing moral distress in end-of-life care and laying a foundation for improved outcomes: an integrative review. J Hospice Palliat Nurs.

[10] Etafa W (2020). Nurses’ knowledge about palliative care and attitude towards end-of-life care in public hospitals in wollega zones: a multicenter cross-sectional study. PLoS ONE.

[11] Epstein EG, Whitehead PB, Prompahakul CH, Thacker LR, Hamric AB (2019). Enhancing understanding of Moral Distress: the measure of Moral Distress for Health Care Professionals. AJOB Empir Bioeth.

[12] Asadi N, Royani Z, Maazallahi M, Salmani F (2021). Being torn by inevitable moral dilemma: experiences of ICU nurses. BMC Med Ethics.

[13] Maffoni M, Sommovigo V, Giardini A, Paolucci S, Setti I (2020). Dealing with ethical issues in rehabilitation medicine: the relationship between managerial support and emotional exhaustion is mediated by moral distress and enhanced by positive affectivity and resilience. J Nurs Adm Manag.

[14] Otaye-Ebede L, Shaffakat S, Foster S (2020). A multilevel model examining the relationships between workplace spirituality, ethical climate and outcomes: a social cognitive theory perspective. J Bus Ethics.

[15] Donkers MA, Gilissen VJ, Candel MJ, van Dijk NM, Kling H, Heijnen-Panis R (2021). Moral distress and ethical climate in intensive care medicine during COVID-19: a nationwide study. BMC Med Ethics.

[16] Corley MC, Elswick RK, Gorman M, Clor T (2001). Development and evaluation of a moral distress scale. J Adv Nurs.

[17] Hariri GR, Yaghmaei F, Zagheri Tafreshi M, Shakeri N (2012). Assessment of some factors related to leave in nurses and their demographic characters in educational hospitals of Shahid Beheshti University of Medical Sciences. J Health Promotion Manage.

[18] Olson LL (2002). Ethical climate as the context for nursing retention. J Ill Nurs.

[19] Abdallah Y (2018). Mortality among very low birth weight infants after hospital discharge in a low resource setting. BMC Pediatr.

[20] Aghaei M, Mohajjel Aghdam A, Bodaghi S, Azami Agdash S (2017). Knowledge and attitude of nurses toward caring for end of life patients. Iran J Nurs.

[21] Bayat M, Shahriyari M, Keshvari M. The relationship between moral distress in nurses and ethical climate in selected hospitals of the iranian social security organization. J Med Ethics Hist Med. 2019;12. 10.18502/jmehm.v12i8.1339 PMC7166247 32328221

[22] Frommelt KH (1991). The effects of death education on nurses’ attitudes toward caring for terminally ill persons and their families. Am J Hosp Palliat Care.

[23] Wenwen Z, Xiaoyan W, Yufang Z, Lifeng C, Congcong S (2018). Moral distress and its influencing factors: a cross-sectional study in China. Nurs Ethics.

[24] Habibzadeh SM, Masouleh ShR, Chehrzad MM, Kazemnezhad Leili E (2020). Moral Distress and related factors in nurses working in Intensive Care Units. J Holist Nurs Midwifery.

[25] Tajnia S, Iranmanesh S, Asadi N, McDermott M (2022). Investigating the effect of inquiry-based stress reduction on mortality awareness and interpersonal problems among intensive care unit nurses. BMC Psychiatry.

[26] Mills M, Cortezzo DE (2020). Moral Distress in the neonatal intensive care unit: what is it, why it happens, and how we can address it. Front Pediatr.

[27] Sauerland J, Marotta K, Peinemann MA, Berndt A, Robichaux C (2015). Assessing and addressing moral distress and ethical climate part II: neonatal and pediatric perspectives. Dimens Crit Care Nurs.

[28] Abate AT, Amdie FZ, Bayu NH, Gebeyehu D, G/Mariam T (2018). Knowledge, attitude and associated factors towards end of life care among nurses’ working in Amhara Referral Hospitals, Northwest Ethiopia: a cross-sectional study. BMC Res Notes.

[29] Asadi N, Salmani F, Asgari N, Salmani M (2022). Alarm fatigue and moral distress in ICU nurses in COVID-19 pandemic. BMC Nurs.

[30] Cerit B, Özveren H (2019). Effect of hospital ethical climate on the nurses’ moral sensitivity. Eur Res J.

[31] Madden JR, Vaughn EA, Northouse B (2015). Benefits of using an early palliative care intervention in pediatric oncology: quality of life and cost savings related to home delivery of cisplatin. J Hosp Palliat Nurs.

[32] Rozman LM, Campolina AG, Lopez RVM (2018). Early palliative care and its impact on end-of-life care for cancer patients in Brazil. J Palliat Med.

[33] Shi H, Shan B, Zheng J, Peng W, Zhang Y, Zhou X, Miao X, Hu X (2019). Knowledge and attitudes toward end-of-life care among community health care providers and its influencing factors in China: a cross-sectional study. Med (Baltim).

[34] Constantina C, Papastavrou E, Charalambous A (2019). Cancer nurses’ perceptions of ethical climate in Greece and Cyprus. Nurs Ethics.

[35] Rautanen A, Baffoe-Mensah F, Zeh H, editors. Sources of Moral Distress Among registered Nurses, 2018.

[36] Lokker M (2018). Palliative sedation and moral distress: a qualitative study of nurses. Applid Nurs Res.

[38] Hamric AB, Blackhall LJ (2007). Nurse-physician perspectives on the care of dying patients in intensive care units: collaboration, moral distress, and ethical climate. Crit Care Med.

[37] Chessa F, Moreno F (2019). Ethical and legal considerations in end-of-Life Care. Prim Care: Clin Office Pract.

[39] Pauly B, Varcoe C, Storch J, Newton L (2009). Registered nurses’ perceptions of moral distress and ethical climate. Nurs Ethics.

[40] Berhie AY, Tezera ZB, Azagew AW (2020). Moral distress and its associated factors among nurses in northwest amhara regional state referral hospitals, Northwest Ethiopia. Psychol Res Behav Manage.

[41] Kadivar M, Zarini P, Moseibi Z, Asghari F, Ranjbar H (2014). Designing and developing an iranian tool to investigate ethical challenges in the intensive care unit. Sci Res J Fac Nurs Midwifery.

[42] Hamric AB (2012). Empirical research on moral distress: issues, challenges, and opportunities. HEC Forum.

[43] Yujeong J, Young-Hae K, Hyunmi S (2019). Ethical climate and Moral Distress among neonatal intensive care unit RNs. Global Health and Nursing.

[44] Esmaelzadeh F, Rajabdizavandi F, Nematollahi M (2023). The relationship between ethical climate and moral distress from the perspective of operating room staff: a correlational study. Clin Ethics.

[45] Hou Y, Timmins F, Zhou Q, Wang J (2021). A cross-sectional exploration of emergency department nurses’ moral distress, ethical climate and nursing practice environment. Int Emerg Nurs.

[46] Fazljoo E, Borhani F, Hoseini SH, Abbaszadeh A (2017). Nurse’s perception of moral distress and ethical climate in the city of Yazd. Med Ethics J.

[47] Asgari S, Shafipour V, Taraghi Z, Yazdani-Charati J (2019). Relationship between moral distress and ethical climate with job satisfaction in nurses. Nurs Ethics.

[48] Prompahakul C, Epstein EG (2020). Moral distress experienced by non-western nurses: an integrative review. Nurs Ethics.

[49] Atashzadeh-Shoorideh F, Tayyar-Iravanlou F, Chashmi ZA, Abdi F, Cisic RS (2021). Factors affecting moral distress in nurses working in intensive care units: a systematic review. Clin Ethics.

[50] Shafiei Z, Norian K, Rafiee Vardanjani L (2020). Association between Nurses’ perception of the ethical climate governing in clinical environment and individual characteristics in teaching hospitals of Shahrekord University of Medical Sciences. IJMEHM.

[51] Zhang N, Gong ZX, Xu Z, Gilal FG (2019). Ethical climate and service behaviours in nurses: the moderating role of employment type. J Adv Nurs.

[52] Hassani M, Sedaqat R, Kazemzadehbeytali M (2017). Correlation between the ethical climate, job stress and job satisfaction in nurses. J Med Ethics.

